# Child Victimization in the Context of Family Violence

**DOI:** 10.3390/ijerph16193569

**Published:** 2019-09-24

**Authors:** Ko Ling Chan

**Affiliations:** Department of Applied Social Sciences, The Hong Kong Polytechnic University, Hong Kong, China; koling.chan@polyu.edu.hk

**Keywords:** child victimization, family violence, cyberbullying, prevention

## Abstract

Child victimization refers to all possible forms of violence experienced by a child. This issue examines multiple types of victimization through a comprehensive approach. To understand child victimization fully, it should be investigated within the context of family violence. The studies in this issue provide evidence of the prevalence of various types of child victimization. As well as child maltreatment and bullying, the emerging form of cyberbullying is examined in several studies. The family has always been the main focus around child victimization, with parenting style as one prominent example. Studies show that some parenting styles are associated with child maltreatment and therefore have suggested that parenting programs may be effective in reducing child victimization. This issue provides up-to-date studies from different regions around the world. It makes a significant contribution to the current debate in child victimization.

## 1. Introduction

Child victimization, which has a broader meaning than child maltreatment, covers various forms of prevalent violence leading to a range of negative impacts on victims. It includes, but is not limited to, child abuse and neglect by parents, peer bullying, child sexual abuse, and exposure to neighborhood violence and crime. Some researchers have also suggested further aspects of victimization, including unintentional injuries by road traffic accidents, drowning, burns, falls, and poisoning that may result from neglect [1].

The term “child victimization” symbolizes a shift in investigating violence against children. In the past, many of the studies of child maltreatment have focused on a single form of child and youth victimization rather than a broad range of child victimization [2]. Studies of a single form of victimization have failed to obtain complete victimization profiles of children and thus have underestimated the impact and range of victimization that children experience. Finkelhor and his affiliates [3] are the first scholars to study polyvictimization or multiple victimization. This is a child-centered approach that studies children’s experience of victimization by looking at multiple types of victimization possibilities, breaking away from boundaries set by academic interest or the scope of child welfare services.

In the last decade, an increasing number of studies have examined child victimization and polyvictimization. This issue covers the most recent studies from countries around the world and addresses specific types of victimization that have drawn academic attention. As well as child maltreatment, this issue includes studies on traditional bullying [4], cyberbullying [5], and both [6,7]. It is clear that cyberbullying has been an emerging social problem in recent years, associated with an exponential growth in social networking. The forms of cyberbullying are evolving including for example sexting [8,9], grooming [8], and doxing [10,11]. 

Student populations have been the major groups targeted in the studies. Nevertheless, several studies illustrate that some vulnerable groups are at higher risk of victimization or poor health, including homosexuals and bisexuals [4], children with Autism Spectrum Disorder (ASD) or Attention Deficit Hyperactivity Disorder (ADHD) [12], children with a disability [13], children affected by migration [14], and delinquent girls [15].

## 2. Family Structure and Child Victimization

The literature has consistently demonstrated that the structure of the family in which children are living has a tremendous effect on their experience of child victimization. Although there have been variations in the definition and conceptualization [16,17,18], family structure is often conceptualized by: (a) the marital status of the child’s biological parents (married, cohabitating, single/separated/divorced, remarried); (b) the child’s living arrangement (whether the child is living with both biological or adoptive parents, with one biological parent and the partner, with a single parent, with other caregivers, living alone or with other children); (c) the number of siblings living in the same household, and (d) the number of other relatives, for example grandparents, in the same household. A traditional family is generally referred to as a family with children living with both biological parents, while a non-traditional family may involve a single parent, or a biological parent and stepparent. In some situations, non-traditional families may refer to those in which children live with neither biological parent (e.g., living with grandparents, relatives, caregivers, children, or alone).

When family structure was defined as the composition of the household where a child was residing, Turner et al. [18] found that the prevalence rates of child victimization varied with the differences in family structure. Children living in non-traditional families (e.g., families with a single parent or stepparent) were more likely to experience victimization than those living in traditional families where two biological or adoptive parents were present. Using a similar definition of family structure, Hanson et al. [17] also showed that the level of exposure to violence increased when adolescents were not living with both of their biological parents. Divergent findings come from other research using different approaches to define family structure. In a study investigating sibling violence [16], family structure was not significantly associated with sibling victimization; what contributed most to sibling victimization was the presence of child maltreatment by the parents. Mixed findings are likely to result when there are variations in the definitions of family structure. Hence, research that carefully defines and conceptualizes the family structure variable is needed before conclusions can be reliably drawn about the effects of family structure on child victimization. 

The family has always been the most significant context affecting child victimization, of which parenting style is one prominent example. It may be oversimplified to consider authoritative parenting techniques, as compared to democratic parenting, as a risk factor. Lo and her affiliates [19] differentiate the effect of authoritarian and authoritative parenting on child maltreatment. They show that authoritarian parenting was associated with all types of child maltreatment, whereas authoritative parenting was associated with a lower risk of all types of child maltreatment. It has been widely agreed that any type of family violence is a risk factor of child victimization [20]. Emery et al. [21] consistently found that partner violence was a significant predictor of child polyvictimization. In my first study of family polyvictimization I indicated that we should put child victimization in the context of family violence [22]. Whilst there are associations between different types of violence within a family, I have also shown that family polyvictimization is associated with extrafamilial violence like bullying and cyberbullying [23]. Any violence prevention policy and program should consider the multiplicity of family violence and how it would impact on child safety and health.

## 3. Implications for Violence Prevention

The WHO [24] published a report on their INSPIRE framework to combat and prevent child abuse and violence. The INSPIRE framework included seven components, namely: Implementation and enforcement of laws, Norms and values, Safe environment, Parent and caregiver support, Income and economic strengthening, Response and support services, and Education and life skills. The framework emphasizes the importance of the involvement of different stakeholders including the government, social service professionals, legal professionals, school professionals, and family. Recognizing the significance of early identification of child abuse to prevent further victimization, the WHO also recommends multidisciplinary collaboration for successful reporting and delivery of services.

The studies included in this issue show various forms of child victimization. We should consider the possibility that a single child could have experienced more than one type of victimization. Having experienced child victimization, the chance of other types of violence taking place in the same family would be greater. Are the existing child protective services capable of addressing the various forms of victimization, including child and family polyvictimization? 

My previous study has provided reliable estimates of child victimization and polyvictimization and examined the associations between victimization and other types of family violence [20]. The strong associations between child victimization and other types of family violence provide strong evidence for the value of proactive screening for other types of violence when one type has been identified. Child protection services should consider all types of victimization that a child may possibly experience [22]. Family disadvantage is a common risk factor for various types of family violence; in particular, low socio-economic status, financial hardship, single parenthood, parental addiction to alcohol or other substances, and poor health. These findings provide evidence to support holistic, whole-family screening to identify at-risk families and should consider incorporating measures to stop other types of violence within a family.

A parenting program is a popular approach in the prevention of child victimization. Evidence shows that parenting programs successfully reduced substantiated and self-reported child maltreatment cases and reduced the potential for child maltreatment [25]. The programs also reduced risk factors and enhanced protective factors associated with child maltreatment. The meta-analytical study published in this issue provides further evidence to support the success of parenting programs in preventing or reducing child maltreatment [26]. Several studies in this issue refer to culture-specific violence prevention programs in Italy [27], Spain [28] and Jamaica [29], contributing scientific evidence to literature which has previously been dominated by studies from North America. 

Victimization and polyvictimization were highly predictive of symptoms of trauma amongst children. Polyvictims were more symptomatic than children who had experienced repeated episodes of the same kind of victimization. Children who experience one kind of victimization are at greater risk of experiencing other forms of victimization. In other words, victimization of any one type left substantial vulnerability for different types of subsequent re-victimization [3]. The researchers in this issue [30] have evaluated the development of trauma-informed screening processes, and evidence-based treatments/trauma focused services. Preliminary evidence shows that trauma-informed approaches have been effective in improving the mental and emotional well-being of children served by community-based child welfare services, as well as their potential for reducing caregiver stress and improving placement stability.

## References

[1] Peden M., Oyegbite K., Ozanne-Smith J., Hyder A.A., Branche C., Rahman A.K.M.F., Rivara F., Bartolomeos K. (2008). World Report on Child Injury Prevention.

[2] Finkelhor D., Ormrod R., Turner H., Hamby S.L. (2005). The victimization of children and youth: A comprehensive, national survey. Child Maltreat..

[3] Finkelhor D., Ormrod R.K., Turner H.A. (2007). Poly-victimization: A neglected component in child victimization. Child Abuse Negl..

[4] Rodríguez-Hidalgo A., Hurtado-Mellado A. (2019). Prevalence and psychosocial predictors of homophobic victimization among adolescents. Int. J. Environ. Res. Public Health.

[5] González-Cabrera J., Tourón J., Machimbarrena J., Gutiérrez-Ortega M., Álvarez-Bardón A., Garaigordobil M. (2019). Cyberbullying in gifted students: Prevalence and psychological well-being in a Spanish sample. Int. J. Environ. Res. Public Health.

[6] Fernández-Antelo I., Cuadrado-Gordillo I. (2019). Moral disengagement as an explanatory factor of the polyvictimization of bullying and cyberbullying. Int. J. Environ. Res. Public Health.

[7] Quintana-Orts C., Rey L. (2018). Traditional bullying, cyberbullying and mental health in early adolescents: Forgiveness as a protective factor of peer victimisation. Int. J. Environ. Res. Public Health.

[8] Machimbarrena J., Calvete E., Fernández-González L., Álvarez-Bardón A., Álvarez-Fernández L., González-Cabrera J. (2018). Internet risks: An overview of victimization in cyberbullying, cyber dating abuse, sexting, online grooming and problematic Internet use. Int. J. Environ. Res. Public Health.

[9] Gassó A., Klettke B., Agustina J., Montiel I. (2019). Sexting, mental health, and victimization among adolescents: A literature review. Int. J. Environ. Res. Public Health.

[10] Chen Q., Chan K.L., Cheung A. (2018). Doxing victimization and emotional problems among secondary school students in Hong Kong. Int. J. Environ. Res. Public Health.

[11] Chen M., Cheung A., Chan K.L. (2019). Doxing: What adolescents look for and their intentions. Int. J. Environ. Res. Public Health.

[12] Hellström L. (2019). A systematic review of polyvictimization among children with Attention Deficit Hyperactivity or Autism Spectrum Disorder. Int. J. Environ. Res. Public Health.

[13] Chan K.L., Lo C.K.M., Ho F., Ip P. (2019). Disability-specific associations with child health and functioning. Int. J. Environ. Res. Public Health.

[14] Chan K.L., Lo R. (2019). Effect of generational status on child well-being: Mediating effects of social support and residential instability. Int. J. Environ. Res. Public Health.

[15] Peterson J., DeHart D., Wright E. (2019). Examining the impact of victimization on girls’ delinquency: A study of direct and indirect effects. Int. J. Environ. Res. Public Health.

[16] Eriksen S., Jensen V. (2006). All in the family? Family environment factors in sibling violence. J. Fam. Violence.

[17] Hanson R.F., Self-Brown S., Fricker-Elhai A.E., Kilpatrick D.G., Saunders B.E., Resnick H.S. (2006). The relations between family environment and violence exposure among youth: Findings from the national survey of adolescents. Child Maltreat..

[18] Turner H.A., Finkelhor D., Hamby S.L., Shattuck A. (2013). Family structure, victimization, and child mental health in a nationally representative sample. Soc. Sci. Med..

[19] Lo C.K.M., Ho F., Wong R., Tung K., Tso W., Ho M., Chow C., Chan K.L., Ip P. (2019). Prevalence of child maltreatment and its association with parenting style: A population study in Hong Kong. Int. J. Environ. Res. Public Health.

[20] Chan K.L. (2014). Child victims and poly-victims in China: Are they more at-risk of family violence?. Child Abuse Negl..

[21] Emery C., Yang H., Kim O., Ko Y. (2019). A multiplicative approach to polyvictimization: A study of intimate partner violence types as risk factors for child polyvictimization in South Korea. Int. J. Environ. Res. Public Health.

[22] Chan K.L. (2017). Family polyvictimization and elevated levels of addiction and psychopathology among parents in a Chinese household sample. J. Interpers. Violence.

[23] Chen Q.Q., Lo C.K.M., Zhu Y., Cheung A., Chan K.L., Ip P. (2018). Family poly-victimization and cyberbullying among adolescents in a Chinese school sample. Child Abuse Negl..

[24] World Health Organization (2016). INSPIRE: Seven Strategies for Ending Violence against Children. https://apps.who.int/iris/bitstream/handle/10665/207717/9789241565356-eng.pdf;jsessionid=07F60BFDB74F2748D901C728271DB110?sequence=1.

[25] Chen M., Chan K.L. (2016). Effects of parenting programs on child maltreatment prevention: A meta-analysis. Trauma Violence Abuse.

[26] Gubbels J., van der Put C., Assink M. (2019). The effectiveness of parent training programs for child maltreatment and their components: A meta-analysis. Int. J. Environ. Res. Public Health.

[27] Palladino B., Nocentini A., Menesini E. (2019). How to stop victims’ suffering? Indirect effects of an anti-bullying program on internalizing symptoms. Int. J. Environ. Res. Public Health.

[28] Pino M., Herruzo J., Herruzo C. (2019). A new intervention procedure for improving classroom behavior of neglected children: Say do say correspondence training. Int. J. Environ. Res. Public Health.

[29] Baker-Henningham H., Scott Y., Bowers M., Francis T. (2019). Evaluation of a violence-prevention programme with Jamaican primary school teachers: A cluster randomised trial. Int. J. Environ. Res. Public Health.

[30] Bunting L., Montgomery L., Mooney S., MacDonald M., Coulter S., Hayes D., Davidson G. (2019). Trauma informed child welfare systems—A rapid evidence review. Int. J. Environ. Res. Public Health.

